# MicroRNA-124 functions as a tumor suppressor and indicates prognosis in human osteosarcoma

**DOI:** 10.3892/etm.2014.2161

**Published:** 2014-12-30

**Authors:** GANG HAN, YAN WANG, WENZHI BI, JINPENG JIA, WEI WANG

**Affiliations:** Department of Orthopedics, General Hospital of PLA, Beijing 100853, P.R. China

**Keywords:** microRNA-124, osteosarcoma, prognosis, proliferation, apoptosis, migration

## Abstract

MicroRNA-124 (miR-124) has been demonstrated to be downregulated in numerous human malignancies and correlated with tumor progression. However, its expression and clinical significance in osteosarcoma remains unclear. Thus, the aim of the present study was to explore the effects of miR-124 in osteosarcoma tumorigenesis and development. Using reverse transcription-quantitative polymerase chain reaction, miR-124 expression was detected in primary osteosarcoma tissues and osteosarcoma cell lines. The correlation of miR-124 expression with clinicopathological factors and prognosis was statistically analyzed. MTT, flow cytometric, and Transwell invasion and migration assays were used to test the proliferation, apoptosis, invasion and migration of osteosarcoma cells transfected with miR-124 mimic. It was found that the expression levels of miR-124 in osteosarcoma tissues were significantly lower than those in corresponding noncancerous bone tissues (P<0.001). In addition, miR-124 downregulation more frequently occurred in osteosarcoma specimens with advanced clinical stage (P<0.001), positive distant metastasis (P=0.005) and poor response to neoadjuvant chemotherapy (P=0.013). Univariate and multivariate analysis identified low miR-124 expression as an unfavorable prognostic factor for overall survival. Furthermore, transfection of miR-124 mimic into MG63 cells was able to reduce cell proliferation, invasion and migration, and promote cell apoptosis. These findings indicate that miR-124 may act not only as a novel diagnostic and prognostic marker, but also as a potential target for the molecular therapy of osteosarcoma.

## Introduction

Osteosarcoma is the most common primary bone tumor in adolescents and young adults (1). Despite progress in therapeutic technologies, including surgery, chemotherapy, radiotherapy and biological therapy, the overall survival (OS) of patients with osteosarcoma remains unsatisfactory. Approximately 80% of patients will eventually develop local relapse or metastatic disease following surgical treatment (2), and pulmonary metastasis is the major cause of fatal outcome (3). Like other malignancies, the development of osteosarcoma is a multistep process with accumulation of genetic and epigenetic changes. However, to date, the highly complex molecular mechanisms underlying its initiation and progression are poorly understood. Therefore, it is necessary to search for novel markers for osteosarcoma, which can accurately identify the biological characteristics of tumors, improve therapeutic strategies and predict clinical outcome.

MicroRNAs (miRNAs) are single-stranded, small noncoding RNAs of 18–25 nucleotides in length (4). They can negatively regulate gene expression through base-pairing to the 3′ untranslated region (3′UTR) of target messenger RNA (mRNA), resulting in translation inhibition or mRNA degradation (5,6). Beyond involvement in diverse biological processes, including cell growth, apoptosis, development, differentiation and endocrine homeostasis (7), emerging evidence strongly suggests that the deregulation or dysfunction of miRNAs contributes to human carcinogenesis and cancer progression (8–10). miRNAs can function as either oncogenes or tumor suppressors according to the roles of their target genes. In terms of osteosarcoma, *in vitro* functional assays have shown that miR-126 and miR-133b inhibit the proliferation, invasion and migration of osteosarcoma cells (11,12). Clinical analysis has demonstrated that decreased miR-145 and increased miRNA-214 expression levels in osteosarcoma are associated with advanced clinical stage and poor prognosis (13,14). Furthermore, Zhou *et al* reported that the upregulation of miR-33a promoted the chemoresistance of MG63 cells to cisplatin (15). These findings indicate that miRNAs may act not only as diagnostic and prognostic markers, but also as potential therapeutic targets of human osteosarcoma.

One of the cancer-related miRNAs is miR-124. It was first reported to be highly expressed in neuronal cells, where it regulates neuronal development and neural plasticity (16). Subsequent studies revealed that miR-124 may modulate the process of tumorigenesis and the behavior of cancer cells. It has been corroborated to be downregulated and exert tumor suppressive function in medulloblastoma (17), breast cancer (18), ovarian cancer (19), cervical cancer (20), gastric cancer (21), colorectal cancer (22), hepatocellular carcinoma (23), pancreatic cancer (24) and prostate cancer (25). However, the expression and function of miR-124 in osteosarcoma is largely unknown. In the current study, miR-124 expression was investigated in paired osteosarcoma and adjacent noncancerous bone tissues by reverse transcription-quantitative polymerase chain reaction (RT-qPCR) assay. The correlation of miR-124 levels with clinicopathological factors and prognosis was also statistically analyzed. Furthermore, the effects of miR-124 on malignant phenotypes of osteosarcoma cells were elucidated.

## Materials and methods

### Patients and tissue samples

This study was approved by the Research Ethics Committee of General Hospital of PLA, (Beijing, China). Written informed consent was obtained from all patients. All specimens were handled and made anonymous according to ethical and legal standards.

A total of 105 primary osteosarcoma and corresponding noncancerous bone tissue samples were collected from the General Hospital of PLA (Beijing, China) for RT-qPCR analysis between March 2002 and February 2008. No patients had previously received a blood transfusion, chemotherapy or radiotherapy. All patients underwent neoadjuvant chemotherapy and wide resection of the tumor. Tumor biopsies were collected prior to neoadjuvant chemotherapy and were fresh-frozen and stored at −80°C. The patient information is summarized in Table I. Clinical tumor stage was classified according to the Enneking staging system (26). Tumor response to pre-operative chemotherapy was assessed using the Huvos grading system (27), on the basis of tumor necrosis in the resected specimen. Good response indicated ≥90% tumor necrosis and poor response indicated <90% tumor necrosis. All of the patients were followed up periodically. The OS time was defined as the time from primary surgery to mortality of the patient or, for living patients, the date of last follow-up.

### Cell culture

Four human osteosarcoma cell lines (MG63, U2OS, Saos-2 and SW1353) and a human normal osteoblastic cell line hFOB 1.19 were purchased from the Institute of Biochemistry and Cell Biology of the Chinese Academy of Sciences (Shanghai, China). Cells were cultured in RPMI-1640 medium (Invitrogen Life Technologies, Gaithersburg, MD, USA) supplemented with 10% fetal bovine serum (FBS), 100 U/ml penicillin, and 100 μg/ml streptomycin in humidified air at 37°C with 5% CO_2_.

### RNA extraction and RT-qPCR

Total RNA was isolated using TRIzol^®^ reagent (Invitrogen Life Technologies, Carlsbad, CA, USA) according to the manufacturer’s instructions. Reverse transcription reaction was carried out using 10 ng total RNA, 50 nmol/l stem-loop RT primer, 1X RT buffer (TIANGEN Biotech Co., Ltd., Beijing, China), 0.25 mmol/l each deoxynucleotide triphosphate (Sigma-Aldrich, Beijing, China), 3.33 U/μl MultiScribe reverse transcriptase (Sigma-Aldrich) and 0.25 U/μl RNase inhibitor (Sigma-Aldrich). The 7.5 μl reaction mixture was initially incubated at 16°C for 30 min, 42°C for 30 min and 85°C for 5 min, and then maintained at 4°C. qPCR was performed using the standard TaqMan MicroRNA assays protocol on an ABI 7500 Real-Time PCR detection system (Applied Biosystems by Life Technologies, Foster City, CA, USA), with cycling conditions of 95°C for 10 min, followed by 40 cycles of 95°C for 15 sec and 60°C for 60 sec. U6 small nuclear RNA was used as an internal control. The RT primers were 5′-GTC GTATCCAGTGCAGGGTCCGAGGTATTCGCACTGGAT ACGACGGCATTCT-3′ for miR-124 and 5′-TGGTGT CGTGGAGTCG-3′ for U6. The PCR primers for mature miR-124 or U6 were designed as follows: miR-124 forward, 5′-GATACTCATAAGGCACGCGG-3′ and reverse, 5′-GTGCAGGGTCCGAGGT-3′. U6 forward, 5′-CTCGCTTCGGCAGCACA-3′ and reverse, 5′-AACGCTTCACGAATTTGCGT-3′. The threshold cycle (Ct) was defined as the fractional cycle number at which the fluorescence passed the fixed threshold. Each sample was measured in triplicate, and the relative amount of miR-124 to U6 was calculated using the equation 2^−ΔCt^, where ΔCT = (CT^miR–124^ − CT^U6^).

### Cell transfection

For RNA transfection, the cells were seeded into each well of 96-well plate and incubated overnight, then transfected with either miR-124 mimic or negative control (NC) (TIANGEN Biotech Co., Ltd.) using Lipofectamine 2000 (Invitrogen Life Technologies) following the manufacturer’s instructions. The sequences of NC were nonhomologous to any human genome sequences, and were used to eliminate potential nonsequence-specific effects. At 48 h after transfection, cells were harvested for further experiments.

### Cell proliferation assay

Cell proliferation capacity was evaluated with an MTT assay. Cells were seeded into 96-well culture plates at a density of 2,000 cells in 200 μl/well and incubated at 37°C for 24 h, after transfection. Then, 100 μl MTT solution (0.5 mg/ml; Sigma-Aldrich, St. Louis, MO, USA) was added to each well, and the cells were incubated for another 4 h. The medium was then replaced with 150 μl DMSO. Spectrometric absorbance at 490 nm was measured using a Multilabel Counter microplate reader (Safire; Tecan Austria GmbH, Grödig, Austria). Cell proliferation was assessed daily for four consecutive days, and the MTT assay was repeated three times.

### Detection of apoptosis by flow cytometry

Apoptosis was detected by flow cytometric analysis. Briefly, the cells were washed and resuspended in 0.5 ml phosphate-buffered saline (pH 8.0) at a concentration of 1×10^6^ cells/ml. Then, the cells were stained with Annexin V and propidium iodide (PI), using the Annexin V Apoptosis Detection kit (TIANGEN Biotech Co., Ltd.). After incubation at room temperature in the dark for 15 min, the cell apoptosis was analyzed on a FACSC LSR II (Becton Dickinson and Co., San Jose, CA, USA).

### Cell migration and invasion assays

The migration and invasion assays were performed using 24-well Transwell chambers (8 μm; Corning Inc., Corning, NY, USA). For the migration assay, 1×10^5^ cells suspended in 200 μl serum-free RPMI-1640 medium were seeded into the upper chamber of the Transwell invasion system, and 500 μl RPMI-1640 medium containing 10% FBS was added to the lower chamber. Following a 24-h-incubation, cells on the upper surface of the membrane were scrubbed off, and the migrated cells were fixed with 95% ethanol and stained with 0.1% crystal violet for 10 min. The number of migrated cells was determined by counting five random fields on each membrane. The invasion assay protocol was similar to that of the migration assay, with the exception that the upper chambers were first covered with 1 mg/ml Matrigel.

### Statistical analysis

SPSS software, version 16.0 for Windows (SPSS Inc., Chicago, IL, USA) was used for statistical analysis. Data are shown as the mean ± standard deviation (SD). The differences between groups were analyzed using the Student’s t-test or Chi-square test. Patient survival and their differences were determined by the Kaplan-Meier method and log-rank test. A Cox’s regression model was used for univariate and multivariate analysis. P<0.05 was considered to indicate a statistically significant result.

## Results

### Decreased expression of miR-124 in osteosarcoma cell lines and primary tumor samples

**Figure 1:**
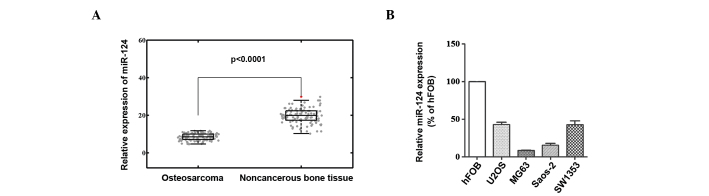
Expression of miR-124 in osteosarcoma tissues and cell lines. (A) miR-124 expression was significantly lower in osteosarcoma tissues than in the corresponding nontumorous samples; (B) miR-124 expression was down-regulated in osteosarcoma cell lines MG63, U2OS, Saos-2, and SW1353, compared to human normal osteoblastic cell line hFOB 1.19. miR-124, microRNA-124.

The expression levels of miR-124 in osteosarcoma tissues, corresponding noncancerous bone biopsy samples, osteosarcoma cell lines and the human normal osteoblastic cell line hFOB 1.19 were detected by RT-qPCR and normalized to U6 small nuclear RNA. As shown in Fig. 1A, the results revealed that miR-124 expression levels were significantly lower in osteosarcoma tissues (8.3±2.1) than in the corresponding noncancerous bone tissues (19.6±4.2; P<0.001). Decreased miR-124 expression was also observed in osteosarcoma cell lines compared with that in hFOB 1.19 cells (Fig. 1B; P<0.001). The MG63 cell line, which possessed the lowest levels of miR-124 expression among all tested osteosarcoma cell lines, was selected for analysis in further experiments.

### miR-124 expression and clinicopathological features in osteosarcoma

The correlations of miR-124 expression with various clinicopathological parameters of osteosarcoma tissues are summarized in Table I. Using the median miR-124 expression in all 105 osteosarcoma patients as a cutoff, the patients were divided into a high miR-124 expression group and a low miR-124 expression group. As shown in Table I, miR-124 was significantly downregulated in patients with osteosarcoma of advanced Enneking stage (P<0.001), positive distant metastasis (P=0.005) and poor response to neoadjuvant chemotherapy (P=0.013). No significant difference was observed between miR-124 expression levels and patient age, gender, tumor size, anatomical location, or the serum levels of lactate dehydrogenase and alkaline phosphatase.

### Correlation between miR-124 expression and prognosis of osteosarcoma patients

**Figure 2:**
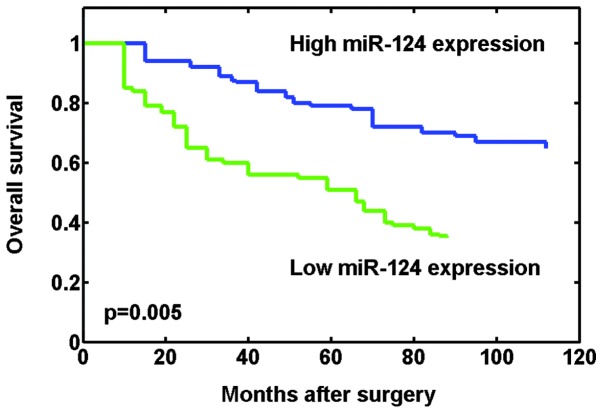
Overall survival curves for two groups defined by low and high expression of miR-124 in osteosarcoma patients. The patients with low miR-124 expression had a significantly shorter overall survival (P<0.001) than those with high miR-124 expression. miR-124, microRNA-124.

Whether miR-124 expression has prognostic potential for the OS of osteosarcoma patients was investigated. Using the Kaplan-Meier method and log-rank test, the OS times of patients with low miR-124 expression levels were found to be significantly shorter than those of patients with high miR-124 expression levels (P=0.005; Fig. 2). In addition, survival benefits were also found in those with smaller tumor size (P=0.034), lower Enneking stage (P<0.001), without metastasis (P=0.011) and a better response to preoperative chemotherapy (P=0.006). Multivariate Cox regression analysis including the aforementioned significant parameters revealed that miR-124 expression [relative risk (RR) 6.325; P=0.015], clinical stage (RR 8.973; P=0.008), metastasis status (RR 3.576; P=0.032), and response to preoperative chemotherapy (RR 4.728; P=0.022) were independent prognostic markers (Table II).

### Effects of miR-124 on cell proliferation, apoptosis, invasion and migration

**Figure 3:**
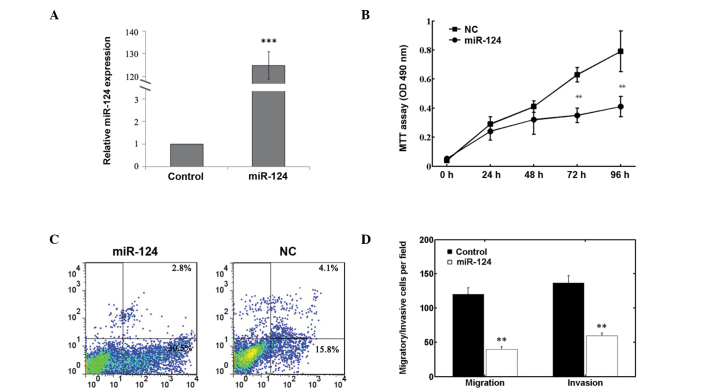
Effects of miR-124 mimics transfection on cell proliferation, apoptosis, invasion, and migration of MG63 cells. (A) The expression level of miR-124 in miR-124 mimics transfected cells was significantly higher compared with NC transfected cells. qRT-PCR was done to detect the expression of miR-124. U6 RNA was used as an internal control; (B) Cell proliferation was measured by MTT assays in MG63 cells transfected with miR-124 mimics or negative control. Data represent the mean ± SD of the experiments performed in triplicate; (C) Apoptosis of MG63 cells was detected by flow cytometric analysis after transfection with miR-124 mimics or negative control; (D) miR-124 suppressed MG63 cell invasion and migration *in vitro*. The Matrigel invasion and migration assays showed that the number of invaded or migrated cells was significantly lower in the miR-124 -transfected group than in the NC-transfected group.

As shown in Fig. 3A, the expression level of miR-124 in the miR-124 mimic-transfected cells was significantly higher compared with that in NC-transfected cells (P<0.01). The MTT assay demonstrated that transfection with miR-124 mimic reduced the proliferation of MG63 cells (Fig. 3B). In addition, promotion of cell apoptosis was also observed in the miR-124 mimic-transfected cells (Fig. 3C). Furthermore, Transwell invasion and migration assays showed a significant reduction in invaded or migrated MG63 cell numbers following miR-124 transfection (Fig. 3D). These results indicate that miR-124 is involved in the negative regulation of osteosarcoma cell growth, invasion and migration *in vitro*.

## Discussion

Dysregulation of miRNAs has been demonstrated to be involved in tumorigenesis and progression in various types of tumor; however, the elucidation of their potential roles in osteosarcoma remains in the early stage of development. In the current study, it was first demonstrated that miR-124 was downregulated in osteosarcoma cell lines and primary tumor samples. Low levels of miR-124 expression were found to be correlated with aggressive clinicopathological features and unfavorable to survival. Furthermore, the transfection of miR-124 mimic into MG63 cells was able to reduce cell proliferation, invasion and migration, and promote cell apoptosis *in vitro*. To the best of the authors’ knowledge, this is the first study regarding the clinical significance and functional attributes of miR-124 in osteosarcoma.

Previous research has reported the tumor suppressive function of miR-124 in numerous human malignancies. *In vitro*, ectopic miR-124 expression inhibits cell growth and induces apoptosis in gastric cancer (21), colorectal cancer (22), pancreatic cancer (24), prostate cancer (28) and cervical cancer (20). The upregulation of miR-124 also reduces cell invasion and migration in ovarian cancer (19), pancreatic cancer (24), hepatocellular carcinoma (23) and breast cancer (18). In addition, miR-124 radiosensitizes human glioma cells (29). *In vivo*, Zhang *et al* revealed decreased miR-124 expression and its association with high tumor grade (Dukes C and D) in colorectal cancer (22). Liang *et al* identified that low miR-124 levels correlated with poor differentiation of breast cancer (18). Zheng *et al* observed that miR-124 downregulation occurred more frequently in hepatocellular carcinoma patients with large tumor size, multiple tumor nodes and advanced tumor stage (23). Moreover, lower expression levels of miR-124 indicated worse prognosis of patients suffering from pancreatic cancer, colorectal cancer or hepatocellular carcinoma (23,24). In xenotransplanted models, miR-124-treated nude mice exhibited smaller tumor sizes and lower tumor weights in comparison with those in the control group (21,22,24,28). These findings suggest that miR-124 might play an important role not only in tumor initiation and progression but also in the molecular-targeted therapy of human malignancies.

The mechanism by which miR-124 expression affects carcinogenesis and cancer development is complex. Some useful targets have been identified during the past few years, including SphK1 (19), STAT3 (22), SLC16A1 (17), Rac1 (24), ROCK2 (23), EZH2 (23), Slug (18), the androgen receptor (28) and CDK4 (29). However, there is no ‘one-to-one’ connection between miRNAs and target mRNAs. An average miRNA can have more than 100 targets (30). Conversely, several miRNAs can converge on a single transcript target (31). Thus, the potential regulatory circuitry afforded by miR-124 may be enormous, and the identification of the complex molecular network involved in its function remains an important subject for future investigation.

In conclusion, the results of the present study revealed that miRNA-124 was downregulated in osteosarcoma cell lines and clinical samples. Low-level expression of miR-124 was significantly associated with a more aggressive and poor prognostic phenotype of patients. Restored miR-124 expression in MG63 cells exhibited antitumor effects *in vitro*. These data suggest an important role of miR-124 in the molecular etiology and gene therapy of osteosarcoma.

## Figures and Tables

**Table I:** Correlation of miR-124 expression with clinicopathological features of osteosarcoma.

		miR-124 expression		
			
Clinicopathological features	Number of cases	High n (%)	Low n (%)	P-value
Age				
<25 years	45	21 (46.7)	24 (53.3)	0.322
≥25 years	60	32 (53.3)	28 (46.7)	
Gender				
Male	57	26 (45.6)	31 (54.4)	0.329
Female	48	27 (56.3)	21 (43.7)	
Tumor size				
>8 cm	55	23 (41.8)	32 (58.2)	0.079
≤8 cm	50	30 (60.0)	20 (40.0)	
Anatomical location				
Tibia/femur	64	29 (45.3)	35 (54.7)	0.231
Elsewhere	41	24 (58.5)	17 (41.5)	
Serum level of lactate dehydrogenase				
Elevated	69	36 (52.2)	33 (47.8)	0.501
Normal	36	17 (47.2)	19 (52.8)	
Serum level of alkaline phosphatase				
Elevated	71	37 (52.1)	34 (47.9)	0.680
Normal	34	16 (47.1)	18 (52.9)	
Clinical stage				
IIA	46	39 (84.8)	7 (15.2)	<0.001
IIB/III	59	14 (23.7)	45 (76.3)	
Distant metastasis				
Absent	74	44 (59.5)	30 (40.5)	0.005
Present	31	9 (29.0)	22 (71.0)	
Response to chemotherapy				
Good	40	31 (77.5)	9 (22.5)	0.013
Poor	65	22 (33.8)	43 (66.2)	

miR-124, microRNA-124.

**Table II:** Univariate and multivariate analysis of overall survival in 105 patients with osteosarcoma.

Variables	Univariate log-rank test (P)	Cox multivariable analysis (P)	Relative risk
MiR-124 expression (high vs. low)	0.005	0.015	6.325
Clinical stage (IIA vs. IIB/III)	<0.001	0.008	8.973
Distant metastasis (absent vs. present)	0.011	0.032	3.576
Tumor size (>8 cm vs. ≤8 cm)	0.034	-	-
Response to chemotherapy (good vs. poor)	0.006	0.022	4.728

miR-124, microRNA-124.
